# Heritability and genetic trend of body weight in dogs of different breeds in Sweden

**DOI:** 10.1093/jas/skad173

**Published:** 2023-05-26

**Authors:** Erling Strandberg, Linda Andersson, Ulf Emanuelson, Charlotte Reinhard Bjørnvad, Sara Ringmark, Åke Hedhammar, Katja Höglund

**Affiliations:** Department of Animal Breeding and Genetics, Swedish University of Agricultural Sciences, 75007 Uppsala, Sweden; Department of Anatomy, Physiology and Biochemistry, Swedish University of Agricultural Sciences, 75007 Uppsala, Sweden; Department of Clinical Sciences, Swedish University of Agricultural Sciences, 75007 Uppsala, Sweden; Department of Veterinary Clinical Sciences, University of Copenhagen, 1870 Frederiksberg, Denmark; Department of Anatomy, Physiology and Biochemistry, Swedish University of Agricultural Sciences, 75007 Uppsala, Sweden; Department of Clinical Sciences, Swedish University of Agricultural Sciences, 75007 Uppsala, Sweden; Department of Anatomy, Physiology and Biochemistry, Swedish University of Agricultural Sciences, 75007 Uppsala, Sweden

**Keywords:** body weight, dog, genetic change, heritability

## Abstract

High body weight (**BW**) in dogs has been associated with developmental as well as degenerative diseases, but the heritability of BW in dog breeds is largely unknown. The aim of the current study was to estimate heritability and genetic change (genetic trend) for BW in a range of dog breeds in Sweden. Body weight registrations from 19 dog breeds (with *n* ranging from 412 to 4,710) of varying body size, type and usage were collected from 2007 to 2016. The average BW of the breeds was 8 to 56 kg. The BW registrations were performed when the dogs were 12 to 24 mo of age (18 to 30 mo for one large-sized breed) in connection with an official radiographic screening program for hip dysplasia. Collected weight records were used to estimate heritability and genetic trends for BW. Several statistical models were used. The preliminary model included the fixed effects of breed (*P* < 0.001), sex (*P* < 0.001), year of screening (*P* < 0.001), litter size (*P* = 0.06), parity of the dam (*P* = 0.03) and linear regression on age at screening (*P* < 0.001), the latter five effects all nested within breed, and the random effects of litter and dam. Season of birth and the quadratic effect of age were also tested, but were not significant (*P* > 0.10). For the genetic analysis, various mixed linear models were tested within breed with different combinations of random effects; the most complex model included random effects of litter, direct additive, and maternal genetic effects, and maternal permanent environmental effects. The average heritability for BW over all 19 breeds was 51%, with a range of 35% to 70%, and the additive genetic coefficient of variance was around 9%. Maternal heritability was 5% to 9% and litter variance was below 10% with one exception (15% in Shetland Sheepdogs). For nine breeds, there was a genetic trend of increasing BW, whereas seven breeds had a genetic trend of decreasing BW. The largest absolute genetic change over a 10-yr period was around 0.6 kg or about 2% of the mean. In conclusion, given the small genetic changes in spite of the high heritability, it seems that there is generally a very weak selection, if any, for BW in the included dog breeds.

## Teaser

Body weight in dogs is highly heritable, but there is very little selection on it.

## Introduction

High body weight (**BW**), due to large body size or excessive body fat content, predisposes for various diseases in dogs (Chiang et al., 2022) including orthopedic diseases, such as hip and elbow dysplasia (Hedhammar et al., 1974; Smith et al., 2001; Sallander et al., 2006). The BW of dogs is affected by both environmental and genetic factors.

The heritability and the genetic variation of a trait provide important knowledge for several purposes. If selection is directly on the individual phenotypic value of BW, then the response to selection (genetic trend) is directly proportional to heritability. If there is indirect selection on BW, by selection on a highly correlated trait, e.g., height, then the selection response in BW is dependent on the heritability of height, the genetic correlation between height and BW, and the genetic standard deviation for BW, given a certain selection intensity. Thus, also in this case, heritability, as a measure of genetic variation, is an important parameter. With higher heritability, we can thus expect a higher genetic change, whether by intent or inadvertently.

Body mass measures, including BW, body mass index (**BMI**), length, and height are known to be highly heritable in most mammals. Heritability (*h*^2^) of height in humans is a classic example, with heritabilities usually being very high, around 0.8 (Yang et al., 2010). Also, human BMI (BW divided by height squared, kg/m^2^) has a high *h*^2^, with reported median 0.75 from twin studies and 0.46 from family-based studies (see review by Elks et al., 2012). There is also a multitude of studies on heritability for BW in farm animals, because this trait is selected for in meat-producing animals (e.g., review by Koots et al., 1994).

In contrast, there are very few studies investigating heritability of BW in dogs (Helmink et al., 2001; Nielen et al., 2001; Schelling et al., 2019), partly owing to lack of reliable BW data. One study has shown that veterinary surgeons in primary practice only infrequently record BW and body conditions during veterinary consultations, potentially partly due to time constraints (German and Morgan, 2008). In Sweden, BW of dogs participating in a routine radiographic hip screening program has been mandatorily recorded since 2005, thus making suitable data available for research.

The aims of this study were, therefore, to estimate heritability and genetic trend for BW in young adult dogs representing a range of dog breeds in Sweden.

## Material and Methods

Data were extracted from the official radiographic screening program for hip dysplasia carried out by the Swedish Kennel Club (**SKK**). In 2005, registration of the dog’s BW was made mandatory in the screening program. According to screening guidelines, official screening results can be obtained from 12 mo of age for most breeds (or 18 mo for certain large breeds). In the present study, screening data registered between January 1, 2007 and December 31, 2016 was used, for dogs born during the years 2005 to 2015 and screened at 12 to 24 mo of age (or 18 to 30 mo for large breeds). Individual dogs without a Swedish registration number at screening, dogs screened prior to the official age and dogs with extreme BW registrations, indicating misreporting, were excluded. If a dog had more than one screening result, the first result was used. Only breeds with a minimum of 15 BW recordings each individual year and a minimum of 400 registrations for the 10-yr period were eligible for inclusion in the study. From breeds meeting these inclusion criteria, 19 breeds were selected to represent a broad range of body size, type and usage in the current study. The breeds represented 8 of 10 breed groups as defined by Fédération Cynologique Internationale (FCI, 2023). Of these breeds, 18 were screened from 12 mo of age, and one (Newfoundland Dogs) from 18 mo of age, according to the program guidelines. The number of records for each breed is shown in Table 1.

**Table 1.** Breeds included in the study, number of dogs (*n*) with body weight (**BW**) records from 2007 to 2016, and average BW (kg) for males and females

Breed	*n*	Average BW	
		Male	Female
Bernese Mountain Dog	3,921	45.7	38.9
Border Collie	2,831	19.1	15.4
Danish-Swedish Farmdog	1,767	9.0	7.2
Finnish Hound	412	27.4	22.6
Flatcoated Retriever	4,710	31.7	26.8
German Shorthaired Pointing Dog	725	29.3	23.8
Irish Soft Coated Wheaten Terrier	554	17.1	14.7
Kleiner Münsterländer	441	22.9	19.1
Lagotto Romagnolo	2,448	15.6	13.1
Malinois	1,235	29.3	24.0
Newfoundland Dog	504	59.7	51.9
Rhodesian Ridgeback	2,039	41.9	34.7
Rottweiler	5,994	43.9	36.5
Shetland Sheepdog	651	8.7	7.5
Staffordshire Bull Terrier	2,109	17.7	14.8
Standard Poodle	1,149	23.1	18.9
Swedish Elkhound	3,884	27.7	23.2
Swedish Vallhund	416	13.0	11.1
Tibetan Terrier	461	11.6	9.5
Total	36,251		

### Statistical analysis

A preliminary statistical analysis was carried out using Proc HPMIXED in SAS (2012), across all breeds, to estimate significance of fixed effects. Results were considered statistically significant at *P* < 0.05. The following statistical model was tested:


$$\begin{array}{l} \begin{array}{ll} & y_{ijklmnpqs}=\;breed_{i}+\;sex_{j(i)}+\;year_{k(i)}+\;season_{l(i)}\\ & \;+\;littersize_{m(i)}+\;parity_{n(i)}+\;b_{1(i)}age\;\\ & \;+\;b_{2(i)}age^{2}+c_{p}+dam_{q}+e_{ijklmnpqs} \end{array} \end{array}$$

where *y*_*ijklmnpqs*_ is the BW at screening, breed_*i*_ is the fixed effect of breed *i* (*i* = 1, …, 19), sex_*j*(*i*)_ is the fixed effect of sex *j* (male or female) within breed *i*; year_*k*(*i*)_ is the fixed effect of year of screening *k* (2007, 2008, …, 2016) within breed *i*; season_l(*i*)_ is the fixed effect of season of birth *l* within breed *i* (March to May, June to August, September to November, and December to February); littersize_*m*(*i*)_ is the fixed effect of litter size class *m* within breed *i* (approximately divided into tertiles within breed); parity_*n*(*i*)_ is the fixed effect of parity *n* of the dam (1, 2, 3+) within breed *i*; b_1(*i*)_ and b_2(*i*)_ are regression coefficients on age and age squared, respectively, within breed *i*; *c*_*p*_ is the random effect of common environment (litter) ~IND(0, $\;\sigma_{c}^{2}$); dam_*q*_ is the random effect of dam ~IND(0, $\;\sigma_{dam}^{2}$); and *e*_ijklmnpqs_ is the random residual ~IND(0, $\;\sigma_{e}^{2}$).

For the genetic analysis the following statistical models were used, within each breed *i*:


$$\begin{array}{ll} y_{jkmnrs}= & \;sex_{j}+\;year_{k}+\;littersize_{m}+\;parity_{n}\\ & +\;b_{1}\;age\;+a_{r}+e_{jkmnrs}, \end{array}$$ [1]

$$\begin{array}{ll} y_{jkmnprs}= & \;sex_{j}+\;year_{k}+\;littersize_{m}+\;parity_{n}+\;b_{1}\;age\;\\ & +c_{p}+a_{r}+e_{jkmnprs}, \end{array}$$ [2]

$$\begin{array}{ll} y_{jkmnpqrs}= & \;sex_{j}+\;year_{k}+\;littersize_{m}+\;parity_{n}+\;b_{1}\;age\;\\ & +c_{p}+dam_{q}+a_{r}+e_{jkmnpqrs}, \end{array}$$ [3]

$$\begin{array}{ll} y_{jkmnpqrs}= & \;sex_{j}+\;year_{k}+\;littersize_{m}+\;parity_{n}+b_{1}\;age\;\\ & +c_{p}+pe_{q}+m_{q}+a_{r}+e_{jkmnpqrs}, \end{array}$$ [4-5]

where all fixed effects are as described for the preliminary model above, *a_r_* is the random additive genetic effect of animal *r* ~ND(**0**, $A\sigma_{a}^{2}$), pe_q_ is the random permanent environmental effect of dam q ~IND(0, $\;\sigma_{pe}^{2}$), *m*_q_ is the random additive maternal genetic effect of dam q ~ND(**0**, $A\sigma_{m}^{2}$), where **A** is the pedigree-based relationship matrix, using all available relationship information from the SKK database. In model [5], the variance-covariance matrix of the animal genetic and the maternal genetic effects was $\left[\begin{array}{ll} \sigma_{a}^{2} & \sigma_{a,m}\\ \sigma_{a,m} & \sigma_{m}^{2} \end{array}\right]$ and, in model [4], the covariance was ignored. Heritability was calculated as $h^{2}=\sigma_{a}^{2}/(\sigma_{a}^{2}+\sigma_{e}^{2})\;$ and coefficient of additive genetic variation (**CV**_**A**_) as additive genetic standard deviation (SD_A_) divided by the mean BW of the breed, where SD_A_ is the additive genetic standard deviation $(\sigma_{a}).$ Variance ratios for litter and dam were calculated with a denominator including all variance components. Maternal heritability from model [4] was calculated as: $m^{2}=\sigma_{m}^{2}/(\sigma_{a}^{2}+\sigma_{m}^{2}+\sigma_{e}^{2})$. Model comparison was done using the Akaike Information Criterion (**AIC**) and the model with the lowest AIC was considered the optimal model. All genetic models ([1] to [5]) were analyzed using the DMU package (Madsen and Jensen, 2013) applying an AI-REML approach.

The genetic trend in BW was calculated as a regression of estimated breeding values on birth year for all dogs born from 2005 to 2015. In order to test whether there was any assortative mating, the correlation between sire BW and dam BW (in mating pairs) was calculated within breed.

## Results and Discussion

### Systematic environmental effects

Breed, sex, year, and the linear regression on age were highly significant (*P* < 0.001) in the preliminary model. Season and the quadratic term for age were nonsignificant (*P* = 0.14 and 0.24, respectively), while parity of dam was significant (*P* = 0.03). Litter size was almost significant (*P* = 0.06) and we decided to include it in the genetic models [1] to [5].

### Model behavior

Model [5] failed to converge or stopped after 200 iterations at or close to the border of the parameter space for the direct-maternal correlation (either +1 or −1) for 10 of the breeds. This was not surprising, as this type of model needs large amounts of data in which both mother and offspring have observed phenotypes. Therefore, model [4], which did not include that correlation, was chosen as the most complex model.

The optimal model (based on AIC) differed between breeds (Table 2). There was a relation between the number of observations for a breed and the optimal model complexity. The four breeds, for which model [1] was optimal, included on average 461 dogs, whereas the six breeds with optimal model [4] included on average 2,958 dogs. Breeds with optimal model [2] and [3] included on average 1,692 and 2,170 dogs, respectively. Thus, it seems likely that a more complex model could be possible to use in the future for some breeds when more data have been accumulated.

**Table 2.** Optimal model^1^, estimates of heritability (*h*^2^, standard error (SE) within brackets), genetic coefficient of variation (CV_A_), litter variance ratio (*c*^2^), variance ratio for dam effect, (dam^2^, model 3) or permanent environmental effect of dam (pe^2^, model 4), maternal heritability (*m*^2^), all in percent, and genetic trend per year from birth year 2005 to 2015 (linear regression, kg/yr; significance: see footnote) for 19 dog breeds

Breed	Model^2^	*h* ^2^ (SE)	CV_A_	*c* ^2^	dam^2^ pe^2^	*m* ^2^	Genetic change per year^3^
Bernese Mountain Dog	4	43 (5)	7.1	4	0	8	0.059****
Border Collie	2	62 (5)	9.1	9			−0.037****
Danish-Swedish Farmdog	2	65 (5)	14.1	3			0.016****
Finnish Hound	1	57 (13)	9.0				0.038****
Flatcoated Retriever	3	43 (4)	7.0	3	7		0.022****
German Shorthaired Pointing Dog	2	45 (11)	6.7	8			0.001^ns^
Irish Soft Coated Wheaten Terrier	1	56 (10)	8.8				0.007*
Kleiner Münsterländer	2	70 (12)	9.6	9			−0.011^ns^
Lagotto Romagnolo	4	54 (5)	10.7	2	0	5	−0.030****
Malinois	4	67 (8)	10.5	0	0	7	0.031****
Newfoundland Dog	2	39 (11)	7.5	9			−0.059****
Rhodesian Ridgeback	4	45 (8)	7.0	0	3	9	0.021****
Rottweiler	4	53 (4)	7.5	7	2	5	0.010***
Shetland Sheepdog	3	35 (12)	14.6	15	0		−0.008****
Staffordshire Bull Terrier	4	54 (5)	7.9	0	0	9	−0.012****
Standard Poodle	3	56 (8)	9.2	1	5		−0.041****
Swedish Elkhound	2	40 (4)	7.4	7			−0.043****
Swedish Vallhund	1	42 (11)	7.1				0.015****
Tibetan Terrier	1	50 (11)	11.4				0.004^ns^

^1^Optimal model based on smallest Akaike’s information criterion.

^2^Model 1 contained only additive genetic effects, model 2 in addition included a litter effect, model 3 in addition included an effect of dam, and model 4 in addition included a genetic maternal effect (but with no covariance between additive direct and maternal genetic effects).

^3^Significantly different from zero *P < 0.05; **P < 0.01, ***P < 0.001, ****P < 0.0001; ns, nonsignificant.

### Heritabilities and additive genetic variation

**Figure 1.**
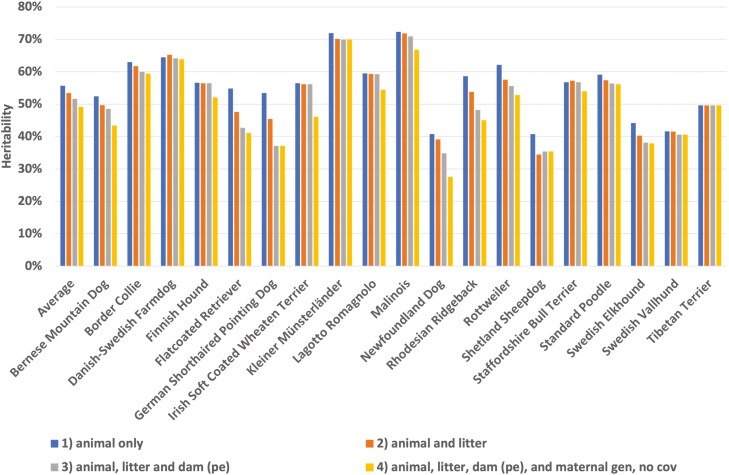
Heritability estimates from 4 models: 1) with random effects of animal only, 2) with random effects of animal and litter, 3) with random effects of animal, litter and dam (permanent environmental effect, pe), and 4) with random effects of animal, litter, dam (pe), and maternal genetic effect (no covariance [cov] between direct and maternal effect), for 19 breeds and averaged over breeds.

Heritabilities from the four models [1] to [4] are summarized in Figure 1. For most breeds, heritability decreased with increasing model complexity. This could indicate that additive variance was somewhat overestimated in the simplest model [1]. On average, the difference in estimated heritability between the simplest and the most complicated model was 7 percentage points, from 56% to 49%.

The optimal model for each breed is presented in Table 2. The average heritability was 51%, however, there was quite large variation between breeds, from 35% to 70%. The Newfoundland Dog and Shetland Sheepdog had heritabilities under 40%, whereas the Border Collie, Danish-Swedish Farmdog, Kleiner Münsterländer, and Malinois had heritabilities over 60%. There was no obvious relation between the average BW of the breed and the heritability estimate (not shown). One reason for the lower heritability for Newfoundland Dogs might be that the lowest age for screening for that breed was 18 mo (as opposed to 12 mo in the other breeds). The longer time until screening might give rise to more environmental influence and thus a lower *h*^2^. On the other hand, the Shetland Sheepdog, which is one of the most light-weight breeds in this study, and is screened from 12 mo, had the lowest heritability of all breeds, at 35%. The reason for this disparity is unknown, but it should be noted that the standard error for the heritability estimate is quite large for both breeds.

**Figure 2.**
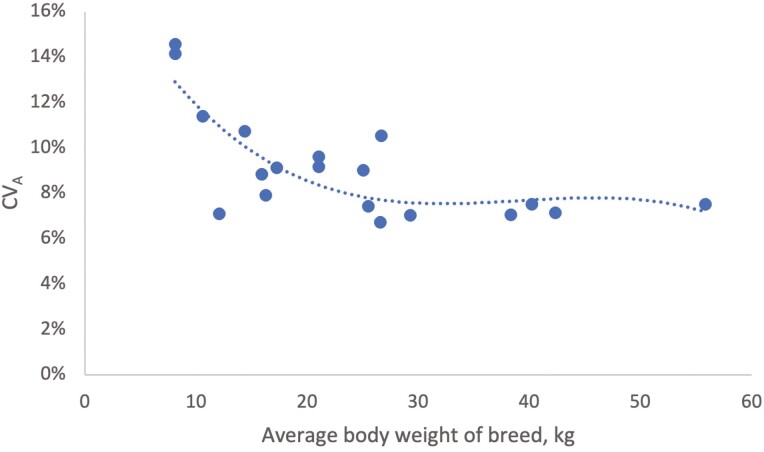
Additive genetic coefficient of variation (CV_A_) as a function of average body weight of breed.

In addition to estimating heritabilities, another way to compare genetic variation between breeds is to use the additive genetic coefficient of variation, CV_A_ (Table 2). On average, CV_A_ was around 9%. However, an increasing CV_A_ with decreasing average BW of the breed was observed from around 20 kg and below, while it was more stable for breeds weighing more than 20 kg (Figure 2). This was particularly clear for Shetland Sheepdog and Danish-Swedish Farmdog, both of which had high CV_A_. However, their heritabilities differed greatly.

As previously mentioned, there are very few published estimates of heritability of BW in dogs. Helmink et al. (2001) reported a heritability of mature BW at 32% to 57% for German Shepherd Dogs and Labrador Retrievers, depending on breed and model used. Nielen et al. (2001) found a much lower heritability of 18% and 8%, the latter with a random litter effect in the model, in a population of Boxers at the age of 2.5 to 3.5 yr. Our estimates ranged from 35% to 70%.

The heritability estimates for BW in the current study were mostly within the range of estimates of heritability of BW for, e.g., beef cattle (Koots et al., 1994). However, CV_A_ was slightly higher than that in dairy or beef cattle, which has been described to be around 6% to 7% (Arango et al., 2002; Lassen and Løvendahl, 2016).

### Maternal and nongenetic effects

The total maternal influence was generally small, between 5% and 12% (Table 2). The maternal effect can be thought of as an unobservable phenotype for maternal ability that affects BW of the offspring. As any phenotype, the effect can be divided into an additive genetic effect (*m* in model [4]) and a permanent nongenetic (environmental) effect of the mother (pe in model [4]). In model [3], dam includes both these components.

Maternal heritabilities (for those breeds with optimal model [4]) were between 5% and 9%. Helmink et al. (2001) also found low maternal heritability (4% to 7%) for mature weight in German Shepherd Dogs and Labrador Retrievers using a similar model as our model [4], but without a permanent environmental effect of dam. Schelling et al. (2019) found a maternal heritability of 22% for birth weight in Labrador Retrievers. It seems logical to expect that the maternal influence is largest for early measures in the offspring, such as birth weight, and that the influence becomes smaller later in life. This was, e.g., shown by Grandinson et al. (2005), where maternal heritability decreased from 19% for birth weight to 6% for 9-wk BW in pigs.

The litter effect, i.e, the effect of the common environment particular for this litter, was generally small (below 10%) and decreased when the dam effect was introduced to the model (results not shown), except for Shetland Sheepdog, for which the effect was stable at ~15%. For several breeds, litter variance became zero when dam was introduced into the model. This could be explained by difficulties in separating these two effects, obviously so for mothers with only one litter, which was between 54% and 82% of all dams, depending on breed. The variance proportion attributable to the permanent environmental effect of dam also decreased when the maternal genetic effect was introduced (results not shown). In several cases, the estimates of litter and dam permanent environmental variance were close to zero, even though the model including that or those effect(s) was the best according to AIC. Thus, for practical purposes, some of these optimal models could be simplified.

### Genetic trend

**Figure 3.**
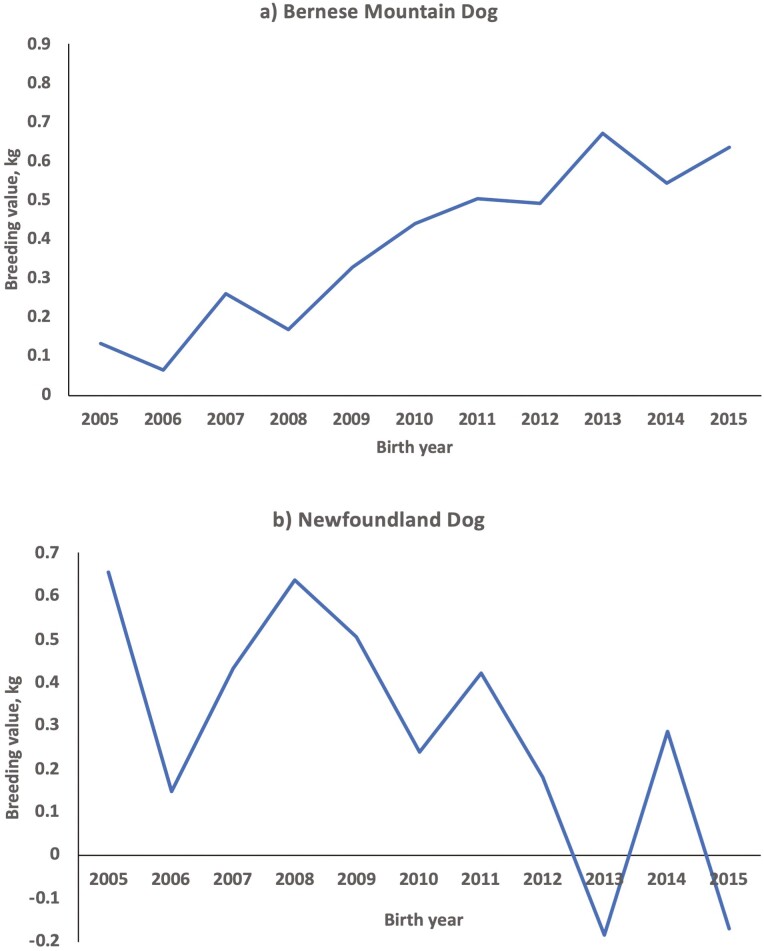
Change in estimated breeding values as function of birth year for a) Bernese Mountain Dog and b) Newfoundland Dog.

The genetic trend of BW was calculated between the birth years 2005 and 2015 (Table 2). For nine of the 19 included breeds there was a genetic trend towards an increasing BW, whereas seven breeds had a genetic trend towards a decreasing BW. For the remaining three breeds, the BW change over time was not significant. The largest annual increase in BW was for Bernese Mountain Dog, Finnish Hound, and Malinois with 0.031 to 0.059 kg/yr, which corresponds to 0.011 to 0.020 SD_A_/yr or 0.12 to 0.15 percentage points of the mean per year. The largest annual decreases were for Newfoundland Dog, Swedish Elkhound, and Standard Poodle with absolute values of 0.041 to 0.059 kg/yr, corresponding to 0.014 to 0.023 SD_A_/yr or 0.10 to 0.19 percentage points of the mean per year. The genetic trend for the two breeds with the largest changes in kg is shown in Figure 3. In species where there is clear selection on growth, e.g., beef cattle, there is much higher genetic change per year. For instance, Sullivan et al. (1999) reported a genetic change of 0.04 to 0.15 SD_A_/yr for weaning (200 d) weight in beef cattle in USA. However, in dog breeds, even if there would be an intention to select for larger (or smaller) dogs, there are generally upper and lower limits, usually on height, that may restrict the change also in BW.

In a previous study, partly using the same dataset, the average breed BW for 72 dog breeds was calculated, including estimates of breed BW changes over the 10-yr period (Andersson et al., 2023). In that study, a change in BW over the 10-yr period was found in 33 of the 72 breeds, with the majority of breeds showing a decrease in BW. For the seven breeds showing a genetic trend towards decreasing BW in the present study (Table 2), six of the breeds also showed a decrease in actual BW in the previous study. In contrast, only two of the nine breeds showing a genetic trend towards increasing BW in the present study also showed an increase in actual BW in the previous study. For Bernese Mountain Dogs and Newfoundland Dogs, the pattern was similar between the studies, with an increase in Bernese Mountain Dogs and a decrease in Newfoundland Dogs (Figure 3) (Andersson et al., 2023).

The BW of an individual is affected by genes as well as environmental factors, such as nutritional status (Hawthorne et al., 2004; Trangerud et al., 2007; Vaysse et al., 2011; Salt et al., 2017). Nutritional status can affect both size and body condition of a young individual, and these factors together with breed and sex, have been associated with adult BW in dogs (Helmink et al., 2000; Hawthorne et al., 2004; Trangerud et al., 2007; Vaysse et al., 2011; Salt et al., 2017). Environmental (nongenetic) factors may also change over time, thus giving rise to a contribution to the total phenotypic trend, that may or may not be in concordance with the genetic trend.

### Concluding remarks

In the present data set, we had access to BW information, while information on size and body composition was ­lacking. It is therefore difficult to discern what constitutes the BW of included dogs. The study population consisted of young adult dogs, with data collected in conjunction with the official screening program for hip dysplasia. In Sweden, dogs traditionally undergo screening at the allowed age for the specific breed or shortly thereafter. Hence, the inclusion criteria for age in our study were decided with the intention to include dogs from young adult age within an age interval when a majority of dogs are examined. Because overweight due to excess body fat seems to be less prevalent in young adult dogs compared to middle-aged or old dogs (McGreevy et al., 2005; Colliard et al., 2006; Lund et al., 2006; Courcier et al., 2010; Mao et al., 2013), the observed BW might be more affected by the dog’s size than by body composition. However, for differentiation, we would have needed access to BCS or Dual energy X-ray absorptiometry, for assessment of body composition (Laflamme, 1997), as well as a measure of size, such as wither height. Longitudinal studies over a broad range of breeds, including measures of BCS and size in addition to BW, are warranted to further explore this matter.

It might be hypothesized that there could be a negative assortative mating for BW (or for correlated size traits), i.e., that heavier males would be mated to lighter females and vice versa, because there are often breed requirements where there are limits on, e.g., wither height. However, the correlations between male and female BW in mating pairs were mostly nonsignificant and those significantly different from zero were positive. The highest values were found for Kleiner Münsterländer (0.34) and other significant values ranged between 0.07 and 0.14, i.e., a sign of positive assortative mating, although not very strong.

Given the estimates of heritabilities, it is possible to calculate a selection intensity *i* that would be needed to result in the observed genetic trend (based on breeder’s equation: Genetic change in SD_A_/yr = (*i h*)/*L*, where *h* is $\surd h^{2}$ and *L* is the generation interval), assuming direct selection on phenotypic value of BW only. For the highest genetic response in SD_A_ (Bernese Mountain Dog), the selection intensity was 0.144 which translates to a proportion selected of 93% (assuming a generation interval of 4 yr). Such small changes, in spite of the high heritability, would indicate a weak selection, if any, for BW in these studied dog breeds.

## Conclusions

The average heritability for BW over all 19 breeds was 51%, with a range from 35% to 70%, and the CV_A_ was around 9%. These values are high enough that any selection, either directly on the phenotype of BW or on a strongly correlated trait, such as height, could result in a large genetic change. Previous selection has at most resulted in a genetic change of 0.6 kg over a 10-yr period or about 2% of the average BW. Given the small genetic changes in spite of the high heritability, it seems that there is generally a very weak selection, if any, for BW in the included dog breeds.

## Abbreviations:

AIC: Akaike information criterion
BMI: body mass index
BW: body weight
CV_A_: coefficient of additive genetic variation
*h* ^2^: heritability
SD_A_: additive genetic standard deviation
SKK: Swedish Kennel Club
